# Influenza and Pneumococcal Vaccination in Hematological Malignancies: a Systematic Review of Efficacy, Effectiveness, and Safety

**DOI:** 10.4084/MJHID.2016.044

**Published:** 2016-09-01

**Authors:** Giuseppe La Torre, Alice Mannocci, Vittoria Colamesta, Valeria D’Egidio, Cristina Sestili, Antonietta Spadea

**Affiliations:** 1 Department of Public Health and Infectious Diseases, Sapienza University of Rome; 2 Local Health Unit Roma 1

## Abstract

**Background:**

The risk of getting influenza and pneumococcal disease is higher in cancer patients, and serum antibody levels tend to be lower in patients with hematological malignancy.

**Objective:**

To assess flu and pneumococcal vaccinations efficacy, effectiveness, and safety in onco-hematological patients.

**Methods:**

Two systematic reviews and possible meta-analysis were conducted to summarize the results of all primary study in the scientific literature about the flu and pneumococcal vaccine in onco-hematological patients. Literature searches were performed using Pub-Med and Scopus databases. StatsDirect 2.8.0 was used for the analysis.

**Results:**

22 and 26 studies were collected respectively for flu and pneumococcal vaccinations. Protection rate of booster dose was 30% (95% CI=6–62%) for H1N1. Pooled prevalence protection rate of H3N2 and B was available for meta-analysis only for first dose, 42.6% (95% CI=23.2 – 63.3 %) and 39.6 % (95% CI=26%–54.1%) for H3N2 and B, respectively. Response rate of booster dose resulted 35% (95% CI=19.7–51.2%) for H1N1, 23% (95% CI=16.6–31.5%) for H3N2, 29% (95% CI=21.3–37%) for B.

**Conclusion:**

Despite the low rate of response, flu, and pneumococcal vaccines are worthwhile for patients with hematological malignancies. Patients undergoing chemotherapy in particular rituximab, splenectomy, transplant recipient had lower and impaired response. No serious adverse events were reported for both vaccines.

## Introduction

### Flu vaccine

Flu is a contagious respiratory illness caused by influenza viruses: influenza A and influenza B viruses infect humans causing widespread, sometimes fatal, disease. Both viruses contain eight gene segments, which encode surface proteins involved in viral attachment, two coat proteins, hemagglutinin (HA) and neuraminidase (NA), on the outer envelope are used to subtype the virus.

Flu viruses are constantly changing, so the vaccine composition is reviewed each year and updated as needed based on which influenza viruses are making people sick, the extent to which those viruses are spreading, and how well the previous season’s vaccine protects against those viruses.

WHO recommends specific vaccine viruses for inclusion in influenza vaccines:

- Trivalent inactivated virus subunit vaccine. HA of H1N1, H3N2, B. One or two doses given at T0 and 3weeks later/a month.
- Inactivated H1N1 v-like virus adjuvanted with AS03. One or two doses given at T0 and 3weeks later/a month.

Some people are at high risk for serious flu complications, thus Health Minister recommends that people aged 65 years and older and everyone aged 6 months through 64 years with chronic diseases (chronic pulmonary, including asthma, cardiovascular, renal, hepatic, neurologic, hematologic, or metabolic disorders, including diabetes mellitus, immunosuppressed, including immunosuppression caused by medications or by human immunodeficiency virus) receive a flu vaccine every year.

Patients undergoing chemotherapy are reported to be at increased risk of contracting, suffering complications and dying from seasonal influenza.^1^ The Centers for Disease Control and Prevention (CDC) recommends annual vaccination for patients on chemotherapy.^2^ However, limited and conflicting data exists to inform the clinician on the efficacy of vaccination programs in this patient population.^3^

Flu vaccines are safe, in fact, most people who get the flu vaccine have no side effects at all: the most common side effects are usually mild and go away on their own.

### Pneumococcal vaccine

Pneumococcal diseases (meningitis, septicemia, pneumonia, sinusitis and otitis media), caused by *Streptococcus pneumoniae,* are a common cause of morbidity and mortality worldwide especially in young children and elderly. Out of over 90 serotypes, only a small minority cause most diseases. At present, there are 3 available pneumococcal vaccines that target either 10, 13 or 23 of the most prevalent serotypes:

- a 23-valent polysaccharide vaccine (PPV23) available since the early 1980s;
- two conjugate vaccines available since 2009, one 10-valent (PCV10) the other 13-valent (PCV13) that gradually replaced the 7-valent conjugate vaccine (PCV7).^4^

The first polysaccharide pneumococcal vaccine was approved in the United States in 1977. It contained purified capsular polysaccharide antigen from 14 different types of pneumococcal bacteria. In 1983, a 23-valent polysaccharide vaccine replaced the 14-valent vaccine.^5^ Ppv23 is used to supplement the immune response following primary vaccination with one of the pneumococcal conjugate vaccines in immunocompromised individuals. Pneumococcal polysaccharide vaccines are associated with poor or absent immunogenicity in children under 2 years of age and failure at any age to induce an anamnestic antibody response upon revaccination. PPV23 is considered safe both regarding severe immediate reactions and potential long-term adverse consequences.^4^

The first pneumococcal conjugate vaccine (PCV7) was licensed in the United States in 2000. In 2010 was approved 10-valent pneumococcal vaccine (PCV10) and a few months later a 13-valent pneumococcal conjugate vaccine (PCV13) was licensed in the United States and Europe.^5^ At present PCV13 is approved as a single dose for the prevention of pneumonia and invasive disease caused by vaccine serotypes of *S. pneumoniae* in all persons, without limits of age. PCV13 is approved for active immunization for the prevention of pneumococcal diseases in infants and children from 6 weeks to 5 years of age, for adults older than 65 years of age or suffering from predisposing medical conditions including chronic diseases of the cardiovascular, bronchopulmonary, liver and renal system, or patients with HIV, diabetes and asplenia.^4^,^6^

In many countries, the routine use of conjugate vaccines has dramatically reduced the incidence of pneumococcal diseases caused by vaccine serotypes included in the vaccines.^4^

The objective of the present study was to perform a systematic review for assessing the efficacy, effectiveness and safety of flu and pneumococcal vaccinations among patients with hematological malignancies.

## Materials and Methods

### Identification of Relevant Studies

This systematic review was performed according to the Preferred Reporting Items for Systematic Reviews and Meta-Analyses (PRISMA) statement.^6^

#### Flu vaccine

The electronic databases PubMed and Scopus were searched, and the following algorithm was applied: (((influenza OR flu*) AND (vaccination OR vaccin*)) AND ((hematological OR hematological) AND (malignanc* OR cancer OR tumor OR neoplasm OR neoplasia)). The search was undertaken in May 2016 concerning papers published from 1 January 2000 to May 2016. Eligible studies were selected through a multi-step approach (title reading, abstract and full-text assessment) by two researchers, working independently.

Furthermore, the references to review, letters, comments, editorials and case reports, identified by the search strategy, were evaluated to add others relevant articles.

#### Pneumococcal vaccine

The bibliographic research was carried out using two medical electronic databases PubMed and Scopus, until April 2016. The research algorithm was: (pneumococc* AND vaccin*) AND ((hematologic* OR haematologic* OR hematopoietic OR haematopoietic) AND (malignanc* OR cancer OR tumor OR neoplasm OR neoplasia)). No restriction of languages or date of publication was applied.

No attempt was made to find unpublished studies.

Furthermore, the references to review, letters, comments, editorials and case reports, identified by the search strategy, were evaluated for retrieving further relevant literature.

### Selection Study and Eligibility Criteria

The first selection was performed filtering duplicate articles by JabRef 2.10 program and ZOTERO 4.0.

The articles identified by search strategy were selected initially analyzing the title and the abstract, independently by two researchers, and then each investigator evaluated the inclusion criteria by full-text. Disagreements between the two reviewers were resolved by a third one.

Articles that take into account efficacy, effectiveness and safety of flu vaccinations among patients with hematological malignancies, were included in the systematic review. Primary study case-control, cohort studies, cross-sectional and clinical trial, were included.

The non-adjuvant, whole-virion vaccination and studies about solid cancer were excluded. Also, when the data on hematological malignancy patients were aggregated with another type of patients, the study was removed.

Only articles published in English, Italian, Spanish were included in the review.

### Data Extraction and Quality Assessment

Data extraction was carried out with the same strategy of the selection of the studies: two researchers collected the data, and the disagreement was resolved by a third researcher.

A quality assessment was performed according to the Newcastle-Ottawa Scale (NOS) for observational studies^7^ and to Jadad scale^8^ for trials.

The following characteristics were collected: first author, study design (cross-sectional, cohort, case-control, RCT, meta-analysis, systematic review), year of publication, country of the first author, quality score, sample size, age of patients, type of diagnosis, type of vaccine, dose (first, booster), adverse events, number of vaccinated patients, type of outcomes (see below), number of patients with outcome.

Concerning the flu vaccination, the following additional information was included: chemotherapy during vaccination (yes/no). Whereas, the pneumococcal vaccination has taken into consideration the timing vaccination after chemotherapy or transplantation or splenectomy.

The characteristics of the study were summarized in tables. The main outcomes considered were:

- Flu vaccine:
  ○ Response rate (seroconversion): defined as the proportion of subjects with an individual 4-fold increase in hemagglutination inhibition (HAI) titer after vaccination;
  ○ Protection rate (seroprotection): hemagglutination-inhibition (HI) antibody titer ≥1:40 following vaccination. The titer represents the level at which approximately 50% of individuals are protected after vaccination;^9^,^10^
  ○ Mean fold increase (MFI), the difference between the log-adjusted geometric mean titers of pre-vaccination and after vaccination.
- Pneumococcal vaccine:
  ○ Efficacy/effectiveness: geometric mean antibody concentrations to different pneumococcal capsular polysaccharide, immunoglobulin concentration or titers before and after vaccination as measure of increase, number of patients with antibody levels in the protective range against various pneumococcal serotype and serum opsonic activity;
  ○ Safety: number of adverse effects registered during the study.

### Statistical analysis

The meta-analysis was realized in the case of the data were homogenous and available.

The meta-analysis was stratified considering: types of flu (H1N1, H2N3, B), dose (1° and booster) and age (adult, children).

The statistical analysis was performed using the software Stats-Direct 2.8.0.

The pooled prevalence with relative 95% confidence interval (95% CI) was calculated and plotted in the forest plot. Cochran Q and I^2^ tests were performed to evaluate the heterogeneity of the studies, using the random-effect model when the test highlighted the differences between studies and the fixed-effect model when no significant differences were shown.^11^ The level of significance was set p < 0.05. The effect size heterogeneity was considered significant when heterogeneity probability values p < 0.05 and I^2^ > 0.20.^12^

The presence of significant heterogeneity was further explored through subgroup analyses.

## Results

### Flu Vaccine

#### Study selection

**Figure 1:**
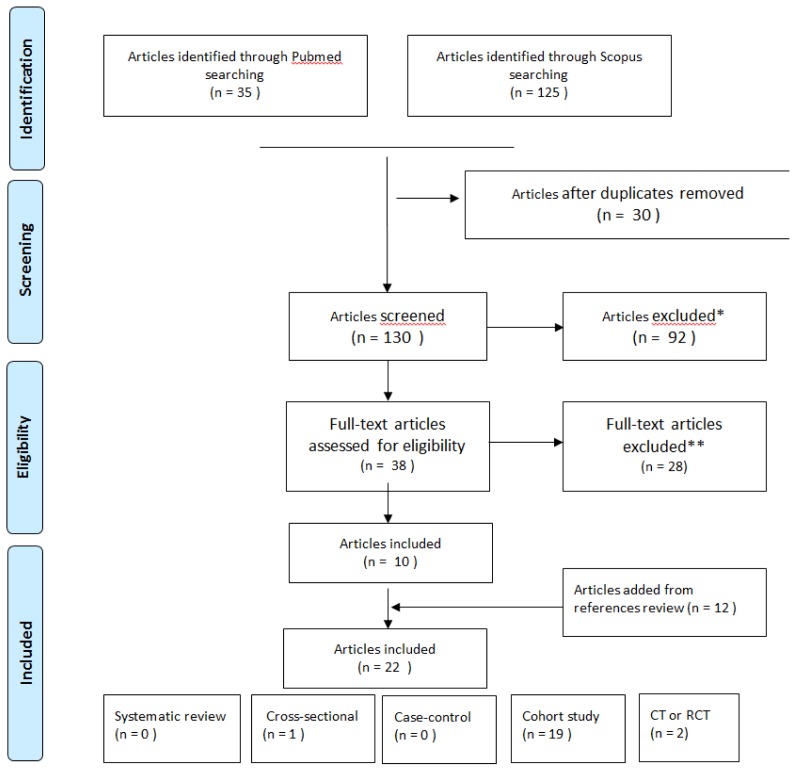
PRISMA Diagram for flu vaccination research strategy. *The papers were removed because they do not consider in the title or the abstract the flu vaccination and/or patients with a hematological tumor. **The papers were removed because they do not respect the inclusion criteria.

The selection of articles is shown in the flowchart, which was performed according to the PRISMA statement (Figure 1). Overall 160 papers were found, 35 articles through Pubmed, 125 through Scopus. Successively, 30 duplicates and 92 articles that did not respect the inclusion criteria were excluded. The remaining papers were analyzed, and from these, 25 articles with no pertinent full text and 2 concerning old vaccine (inactivated influenza A/New Jersey/76 whole virus vaccine) were removed too. Twelve papers were added from the references of the papers collected.

For the analysis, 22 papers were finally selected: 19 cohort studies, 2 RCT, 1 cross-sectional.^13^–^34^

#### Characteristics of the studies

The characteristics of the studies included are shown in Table 1.

In particular two trials investigated effectiveness and safety of flu vaccine in onco - hematologic patients. In Monkman et al.^33^ studies, the rate of seroconversion among vaccinated patients (21%) was significantly higher than that in unvaccinated patients (0%; p<0.001). Instead, there were no significant differences in the geometric mean titers or rates of seroprotection between the vaccinated and unvaccinated groups and there were no differences in the rates of response to the vaccine between patients on or off chemotherapy, on or off rituximab.

In Dignani et al.^34^, most patients (47) had onco-hematological cancers and (18) had solid tumors, in all patients with a diagnosis of influenza H1N1 the 30-day mortality was measured. This was 0% in 19 vaccinated patients, and 27% (12/45) in non-vaccinated patients: all deaths occurred among the non-vaccinated patients.

In an other cohort study Safdar et al. dealt of purified and recombinant DNA (rDNA), they have been employed to obtain an increasing dosage of HA vaccine 15, 45, or 135 mg of each HA. There was a trend toward an increased antibody response frequency in the higher rHAO dose groups. The response frequencies were higher for A/H3 and A/H1 than for standard dose but not for influenza B; however, none of the differences were statistically significant.^26^

In Esposito et al. children with a diagnosis of onco-hematological disease were enrolled: influenza vaccination was effective in reducing influenza-related morbidity among all of the vaccinated children, regardless of the time since their last cancer therapy (for <6 months or off therapy for 6–24 months), one of the major benefits was the reduction in the number of hospitalizations. The effectiveness of vaccination in decreasing the number of upper respiratory tract infection and lower respiratory tract infection, days of fever, antibiotic courses, and lost school days was greater in the children who had been off therapy for less than 6 months.^29^

The safety and tolerability of the vaccine were excellent after both doses.

The adverse events (AEs) are shown in Table 2: only a minority of patient experienced AEs regardless of the time since the completion of cancer therapy or vaccine dose, and none of the AEs were serious; most of them were mild and did not require treatment.

Only one study shod the data concerning the mean fold increase (MFI). The authors referred mean ±SD (range): 0.26±0.33 (0–1.00) for H1N1, 0.17±0.34 (0–1.00) for H3N2, 0.35±0.34 (0–1.20) for B serotype.^20^

#### Pooled analysis

The meta-analysis on response rate and protection rate were carried out grouping by serotypes of vaccine (H1N1, H3N2, B). A sensitivity analysis was added stratify by age group (children and adults). Figures 2a and 2b showed the forest plots on adults.

In relation to protection rate of H1N1 first dose, the pooled prevalence resulted 31% (95% CI=18.3 – 45.3%), with a Cochran Q=182.97 (df=15), p < 0.0001, so a random-effects model was applied. In addition, considering the booster dose of protection rate, the pooled prevalence for H1N1 was 30% (95% CI=6–62%), with a Cochran Q=80.73 (df=4), p < 0.0001 (Figure 2a).

Protection rate of H3N2 first dose resulted in a pooled prevalence of 42.6% (95% CI=23.2 – 63.3%), Cochran Q=62.16 (df=6), p < 0.0001. Protection rate of B first dose resulted in a pooled prevalence 39.6 % (95% CI=26%–54.1%), Cochran Q=30.25 (df=6), p < 0.0001.

The meta-analysis of protection rate for booster doses H3N2 and B was not realized because only one study reported results with rates respectively 2/20 and 6/20.^20^

Considering the response rate outcome the pooled prevalence for the first dose of H1N1 resulted 30% (95% CI=21.8–39.1%), Cochran Q=94.47 (df=17), p < 0.0001, for booster dose 35% (95% CI=19.8–51.2%), Cochran Q=65.55 (df=7), p < 0.0001 (a random effect model was applied) (Figure 2b).

Response rate of H3N2: for one dose the pooled prevalence was 21.7% (95% CI=11.1–34.8 %), Cochran Q=52.30 (df=8), p < 0.0001, and for the booster dose was 24% (95% CI=17–32%), Cochran Q=0.87479 (df=2), p=0.64 using a fixed effect model (Figure 2b).

Response rate for B: for one dose the pooled prevalence was 23.6% (95% CI=11.9–37.8%), Cochran Q=58,837007 (df=8), p < 0.0001, and after booster dose 29% (95% CI=21–37%), Cochran Q=0.71231 (df=2), p=0.7004 applying a fixed effect model (Figure 2b).

**Figure 3:**
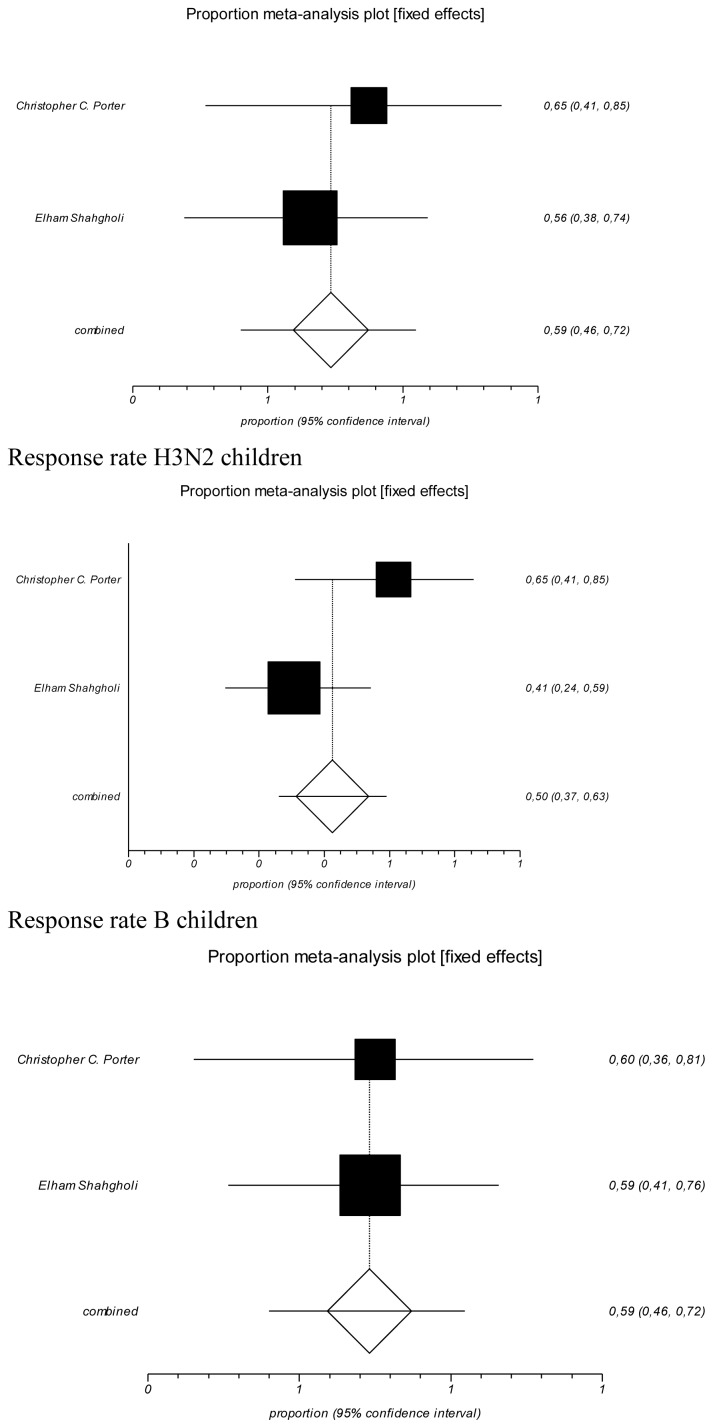
Forrest plots of Response Rate stratify by serotype of vaccine (children) independently from the dose (first or booster). Response rate H1N1 children

Figure 3 showed the forest plot of the cohort studies focused on children setting. In relation to response rate in children, the pooled prevalence resulted in a value for H1N1 one dose of 59.3% (95% CI=46–71.9 %), Cochran Q=0.36 (df=1), p=0.546; for H3N2 a value of 50% (95% CI=36.8–63.2%), Cochran Q=2.87 (df=1), p=0.09; for B a value of 59.2% (95% CI=45.9–71.8%), Cochran Q=0.001 (df=1), p=0.9744. The all three were with fixed effect model.

Protection rate in children using the data published by Shahgholi et al. was 34% (11/32) for H1N1, 21.8% (7/32) for H3N2, 25% (8/32) for B.^31^

As regards the recombinant vaccine response frequency and the highest mean titer for each of the 3 viruses was the highest dose group (135 mg). 40% of patients who had received 45 mg of rHAO and 60% who had received 135 mg of rHAO exhibited an increase in influenza H3N2 neutralizing antibody titer. For influenza H1N1, 67% of patients who received 135 mg of rHAO developed neutralizing antibody titer. Neutralizing antibody responses for influenza B were not different in either recipients of standard vaccine (40%) or those who received 135 mg of rHAO (50%).^26^

### Pneumococcal Vaccine

#### Study selection

**Figure 4:**
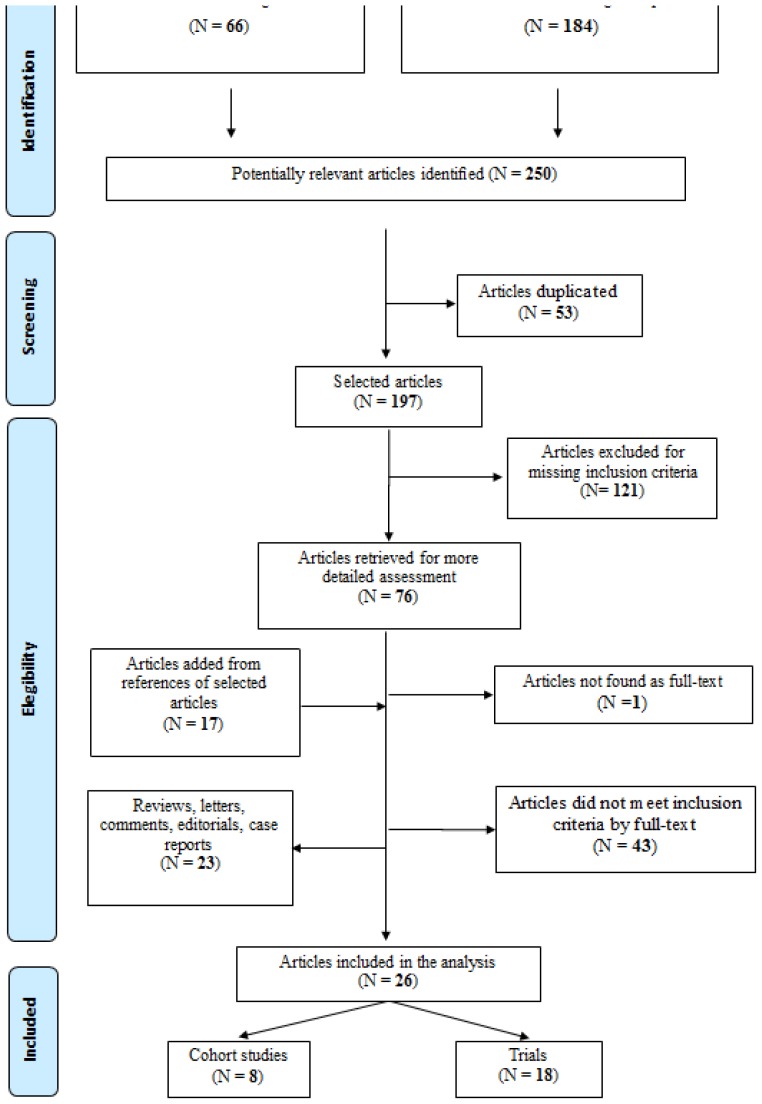
Flow-chart of pneumococcal research strategy.

Figure 4 shows the flow-chart of bibliographic search. The search strategies identified 250 articles (66 PubMed records and 184 Scopus records). After the exclusion of duplicates, 197 articles were selected. After analyzing title and abstract, 121 studies were deleted. Full texts were obtained for the 76 remaining articles, and 9 papers were eligible for inclusion in the review.

Also, 17 papers were added from the analysis of the references to selected articles.

Finally, 26 studies were included in the systematic review, of which 18 trials and 8 cohort studies.^35^–^60^

#### Characteristics of the studies

The main characteristics of included studies are reported in the Tables 3 and 4. The first study was published in 1978^58^ and the last one was published in 2015.^57^

Regarding geographical distribution, 5 cohort studies and 14 trials were conducted in Europe, 2 cohort studies and 4 trials in America and 1 cohort study in Asia.

Concerning the age of the population studied: 2 cohort studies^38^,^51^ and 3 trials^35^,^41^,^54^ were conducted exclusively among children patients.

About diagnosis, 4 articles focused on patients with chronic lymphocytic leukemia,^44^,^53^,^59^,^60^ three Hodgkin’s disease,^37^,^42^,^58^ two papers multiple myeloma,^45^,^56^ one non-Hodgkin’s disease patients^55^ and 1 acute lymphocytic leukemia.^41^

The examined pneumococcal type of vaccine were: 14-valent, 23-valent polysaccharide, 7-valent conjugate and 13-valent conjugate.

As far as concerns the outcomes, all papers evaluated the efficacy/effectiveness and only 2 cohorts^37^,^50^ and 3 trials^43^,^54^,^55^ reported data about safety.

#### Description of results of included studies

Eight cohort studies investigated effectiveness and safety of pneumococcal vaccine exclusively in onco - hematologic patients. Others articles in literature considered in the results also patient with hematologic disorder non-neoplastic.

The older study is the work by Braconier about 14 valent vaccine.^36^ The authors evaluate serum opsonic activity and antibody responses to 3 pneumococcal polysaccharide antigens in patients with hematologic malignancies splenectomized or not. They found that only patients with Hodgkin’s disease have a reduced immunization response. The other studies, except for Shah et al., evaluated the effectiveness 7 valent and 23 valent.^57^

Three papers studied 23 valent in patient with hematological malignancies splenectomized and not: Cherif et al. evaluate a cohort of the splenectomized patient for hematological malignancies.^39^ Observing levels of pneumococcal polysaccharide antibodies they found that 28% mounted a poor pneumococcal polysaccharide antibody response and remained at risk for pneumococcal infections despite vaccination. Median age at vaccination was significantly higher in poor responder: Hinge et al. considered the geometric mean antibody titer in patients with multiple myelomas treated with high-dose melphalan with autologous stem cell support.^45^ They found that 33% of the patients responded to the vaccine. They also found a statistic significant association between response to the vaccine and disease stage (p=0.01). They conclude that vaccination against *S. pneumoniae* before autologous stem cell transplantation is reasonable at least in patients responding well to induction therapy, but it is important to be aware that the response is frequently poor, and the duration of it is unknown. Llupià et al. evaluate seroconversion in a splenectomized patient.^47^ The proportion of responders was 70%. Immunosuppression and the reason for splenectomy (hematologic neoplasia versus non-malignant hematologic diseases) were independent predictors of non-response to vaccination. The OR of non-response to vaccination in patients with hematologic neoplasia compared with patients with non-malignant hematologic diseases was 7.37.

Two publications had analyzed the effect of 7 valent and 23 valent. The first one, Pao et al.,^51^ retrospectively analyzed the response of allogeneic hematopoietic cell transplantation (HCT) recipient. Three PCV-7 were administered in the majority of patients when minimal milestones of immune reconstitution were achieved. In total, 62% of patients responded to PCV-7 (45 out of 51 children; 34 out of 76 adults; p<0.001). Individuals older than 50 years responded significantly better if vaccinated following the acquisition of specific minimal milestones of immune competence. Twenty-seven patients who did not respond to an initial series of PNCRM7 were subsequently vaccinated with PPV23 or received a second series of PNCRM7. Patients were vaccinated at a median of 270 days following their last pneumococcal vaccine. 25% of patients responded to PPV23 and 7 to the second series of PNCRM7 (p=0.06). The second one, Meerveld-Eggink et al.,^48^ published on vaccine response in patients following reduced intensity conditioning. The pcv-7 response was measured in patients conditioned with fludarabine and cyclophosphamide and 200 cGy of single-dose total body irradiation, or fludarabine and 200 cGy of total body irradiation. Patients were immunized if they were a minimum of 12 months post-HCT and no longer required immunosuppressive therapy. The median time to first vaccination was 15 months. Following the second PCV-7, 73% of patients responded to all seven serotypes, except serotype 6B. They conclude that vaccination of patients at a median of 15 months post-allo-RIST leads to significant rise in concentrations of pneumococcal, Hib, and TT antibodies in the majority of patients.

In a recent study, Shah et al. analyzed response rate (seroconversion in a seronegative individual,^57^ a 3-fold geometric mean fold rise of the IgG geometric mean concentration consecutive) cord blood transplant recipients (CBT) treated for hematological malignancies (predominantly acute leukemia) from October 2005 to February 2012. Patients received Prevenar 7, Prevenar 13 or both about 17 months post-CB. 53% responded to all 3 clinically critical pneumococcal serotypes (14, 19F, and 23F). Response rates by clinically significant serotype did not differ between children (60%) and adults (49%). Among the 28 non-responders, 33% responded to 1 to 2 critical serotypes and 9 patients (16%) did not respond to any. This study demonstrates that CBT recipients can respond to protein conjugated vaccines similar to adult donor allograft recipients. This study concludes that in patients off immunosuppression therapy in whom responses to protein conjugated vaccines were documented, live vaccines are safe and can be effective. The sample size of this study was relatively small. Cheng et al. had evaluated antibody response to PCV 7 in a population of pediatric patients,^38^ with a median age of 9.5 years. They found that after two doses of PCV-7, 86–100% of patients had protective antibody titers against the seven vaccine serotypes. The authors concluded that PCV could elicit protective anti-pneumococcal antibody responses in pediatric oncology patients.

Concerning the trials, the older one^58^ evaluated the impaired effectiveness of the dodeca-valent vaccine, three weeks after the immunization, in patients with Hodgkin’s disease treated with subtotal radiation or chemotherapy in terms geometric-mean of antibody concentration.

The effectiveness of 14 –valent was analyzed in children affected by Acute Lymphocytic Leukemia (ALL) and adults with early-stage Hodgkin’s disease.^41^,^42^ Both studies found that the effectiveness of the vaccine is reduced during the therapy. Feldman et al. also found that after 6 months, only a few patients maintained a protective level of antibody response.

Most of the studies evaluate the effectiveness of 23 valent.^40^,^43^,^44^,^46^,^49^,^50^,^52^,^55^,^56^,^59^ All of these studies were conducted on adults. Parkkali and colleagues found that in allogeneic Bone Marrow Transplantation (BMT) recipients the response at 1 month after vaccination was poor and similar in the late (18–20 months after BMT) and early (6 months) vaccination groups.^52^ However, two-fold responses in the concentration of antibodies to the most immunogenic Pnc serotype 3 occurred more frequently in the late group. They conclude that Pnc vaccines should not be given later than 6–8 months post-BMT. Petrash et al. found that vaccination with pneumococcal polysaccharides in splenectomized patients with non-Hodgkin lymphoma (NHL) elicits an adequate antibody response in 45.4% of the cases and should be administered.^55^ Revaccination of the nonresponders does not further increase the pneumococcal antibody levels. Robertson et al. in multiple myeloma patients obtain that 40% of them achieved protective specific antibodies 4–6 weeks following vaccination. In 26 (61%), however, suboptimal titers were reached, and in 13 patients (30%) antibody titers remained below the 10th centile. This study confirms that patients with multiple myeloma have impaired the ability to mount a good humoral response to vaccination. Gandhi et al.^43^ in the prospective study compare serological responses to pneumococcal polysaccharide and another vaccine between autoPBSCT (peripheral blood stem cell) and auto and alloBMT recipients. They found no significant difference between transplant categories, or between healthy controls. Total lymphocyte counts were significantly reduced in the autoPBSCT and autoBMT but not in alloBMT cohorts compared to controls. Hartkamp and colleagues studied patient with CLL.^43^ After vaccination, the number of pts with Ab levels in the protective range against pneumococcal serotypes increased from 9 (38%) to 12 (50%) of 24 patients. Nodoy 49 studying patients with malignant lymphoma, years after ABMT, found that the response to the pneumococcal vaccine was reduced in respect of the control group. In another study found that a larger proportion of patients with solid tumors (81%) than lymphoma (38%) achieved protection. The same author in a further publication^50^ evaluated if patients with solid tumors and malignant lymphoma undergoing chemotherapy would respond serologically to vaccination against influenza and pneumococcal disease. The results show that higher proportion of patients with solid tumors (81%) than lymphoma (38%) achieved protection. Age, months on chemotherapy, and curative versus palliative treatment did not influence responses to vaccination. After vaccination with a 23-valent polysaccharide vaccine against pneumococci, most patients and controls achieved protective serum levels of antibodies against the different serotypes. The responses in controls were, however, generally stronger to all serotypes. Tumor type did not influence this vaccination response. They conclude that cancer patients achieved adequate responses to influenza virus and *Streptococcus pneumoniae.* These are not live vaccines and are therefore safe for immunocompromised patients. Routine vaccinations against influenza virus and *Streptococcus pneumoniae* should be considered in cancer patients undergoing mild to moderately immunosuppressive chemotherapy.

In a study Sinisalo et al. found that antibody response rate to vaccination against pneumococcal polysaccharide was lower in patients with CLL than in controls. In the patients’ group, clear evidence for a good responsiveness was detected only in the case of *Hemophilus influenzae* B (Hib) conjugate antigen. In conclusion, plain polysaccharide vaccines seem to be ineffective in patients with CLL, whereas conjugate vaccines are immunogenic and may offer protection against infections caused by encapsulated bacteria in these patients.^59^

On the other hand, Landgren et al. obtained a significant response to primary vaccination with the same pneumococcal capsular polysaccharide vaccine as well as on two revaccination occasions in splenectomized patients either for trauma or Hodgkin lymphoma.^45^ Eisenberg et al. recorded significant differences in antibody titer increase between splenectomized patient for trauma (T) or hematologic malignancies (HM) in response to the 23-valent polysaccharide vaccine.^39^ In the HM group, only 8/23 and 6/23 showed a titer increase of twice or more the base value for IgG and IgM respectively, whereas an adequate response was shown by 16/21 and 16/20 respectively in the trauma group.

Two investigations evaluate the effectiveness of 23 valent and 7 valent vaccines. Chan et al. evaluate previously treated HD patients immunized with 7-valent pneumococcal conjugate vaccine followed by one dose of 23-valent polysaccharide pneumococcal vaccine.^36^ To determine the priming effect of the 7-valent vaccine, they measured the antibody response to six serotypes contained in both vaccines in HD patients who received either both vaccines or the 23-PS vaccine only. They recorded a geometric mean antibody concentration after immunization with 23-PS vaccine significantly higher for five of the six measured serotypes in HD patients primed with 7-0MPC vaccine compared with responses in HD patients who received 23-PSvaccine only. Patel et al.^54^ recruited children underwent autologous or allogeneic hematopoietic stem cell transplantation for malignant diseases. They received 2 doses of PCV7 followed by 1 dose of Pn-PS23 or of 1 dose of Pn-PS23 followed by an additional dose of Pn-PS23. After administration of the booster dose of pneumococcal polysaccharide vaccine in previous conjugate recipients, very high concentrations against all PCV7 serotypes were achieved. Other two articles evaluated 7-valent vaccine. Antin et al.^34^ considered in their study patients who underwent autologous Hematopoietic Stem Cell Transplantation for hematologic malignancies immunized with PCV7 at 3, 6, and 12 months. After the 3-dose series of PCV7 after autoHCT, more than 60% of the study patients had protective concentrations of antibody to all 7 vaccine serotypes regardless of immunization before stem cell collection. Sinislo et al. evaluated response to the 7-valent conjugated pneumococcal vaccine in patients with chronic lymphocytic leukemia.^60^ Antibody response rates to vaccine antigens were lower in patients with CLL than in controls; however, when the vaccine was administered before chemotherapy and development of hypogammaglobulinemia, a significant response to at least six antigens was obtained in almost 40% of the CLL patients. After vaccination, the antibody concentrations were significantly lower in CLL patients than in the controls for all serotypes.

Pasiarsky and colleagues in a recent work analyze antibody and plasmablast response to 13–valent pneumococcal conjugate vaccine in Chronic Lymphocytic Leukemia Patients.^53^ They evaluated levels of specific pneumococcal antibodies, the levels of IgG and IgG subclasses and selected peripheral blood lymphocyte subpopulations including the frequency of plasmablasts before and after immunization. An adequate response to vaccination, defined as an at least two-fold increase in specific pneumococcal antibody titers versus pre-vaccination baseline titers, was found in 58.3% of CLL patients and 100% of healthy subjects. Both the CLL group and the control group demonstrated a statistically significant increase in the IgG2 subclass levels following vaccination (P50.0301). After vaccination, the proportion of plasmablasts was significantly lower (p<0.0001) in CLL patients in comparison to that in controls. Patients who responded to vaccination had a lower clinical stage of CLL as well as higher total IgG, and IgG2 subclass levels. No significant vaccine-related side effects were observed. PCV13 vaccination in CLL patients is safe and induces an effective immune response in a considerable proportion of patients. To induce an optimal vaccination response, the vaccine PCV13 should be given soon after CLL diagnosis.

## Discussion

### Flu

Studies measuring immunogenicity following vaccination with a single dose of pH1N1 vaccine in the general population showed protection rates over 85%.^61^

On the other hand, in patients with tumor, in particular during the active phase of cancer treatment, many components of the B- and T-immune system are deficient;^62^ the risk of getting influenza infection is higher in cancer patients than in healthy population, with an estimated age-specific rates for influenza-related hospitalization and death of 219 and 17.4 per 100,000, respectively, for patients age <65 years, and of 623 and 59.4 for patients age ≥65 years. Rates are lowest in patients with hematological malignancy with serum antibody levels tending to be lower than those observed in patients with solid tumors; the clinical significance of this is unknown.^23^

The aim of this paper was to summarize the results of all primary studies in the scientific literature about the protection and response rate of flu vaccine in onco-hematological patients, and this was done through a metanalysis both for children and adult population.

About protection rate of H1N1 in the adult setting, the pooled prevalence was not increased between first dose and booster and it was about 30%. Protection rate of H3N2 resulted in a pooled prevalence of 42.6%. Protection rate of B first dose resulted in a pooled prevalence 39.6 %.

About the response rate, the pooled prevalence resulted in value for H1N1 of 30% to 35% for a booster dose. For H3N2 a value of 21.7% to 23%, for B a value of 23.6% to of 29% for a booster dose. For all serotypes, the response rate was increased by a booster dose.

About protection rate in pre-vaccine children, Shahgholi et al. reported a proportion of 11/32 (34%) for H1N1, 7/32 (21.8%)for H3N2, 8/32 (25%) for B with protective titer.^32^ After vaccination, the pooled prevalence resulted, for H1N1 one dose 59.3%, for H3N2 50%, for B 59.2%. This immune response is comparable to control at least for H3N2.

The study of Bate of children with cancer in the United Kingdom demonstrates a limited but acceptable response to a pandemic (H1N1) 2009 vaccine; a higher proportion of children with solid tumors, compared with those with hematological malignancies, achieved a 4-fold increase in HAI titers. The data suggest that AS03b-adjuvanted (H1N1) 2009 vaccine can induce limited but useful protective immune response in children with cancer.^22^

The immunologic response to trivalent inactivated influenza vaccine in children receiving maintenance chemotherapy for ALL was less than that seen in healthy children. Nonetheless, a significant percentage of children with ALL had 4-fold rises in HAI antibody titers. Until data demonstrate the efficacy of the influenza vaccine in this patient population, clinicians should immunize these high-risk children as well as all of their household contacts greater than 6 months of age on a yearly basis.^31^

Overall the vaccine resulted in a low but measurable rate of seroconversion in patients with hematological malignancies, despite this low rate of response, the influenza vaccine is likely still worthwhile for patients with hematological malignancies, as it is an inexpensive intervention with few side effects. However, physicians and patients should be aware that vaccination does not eliminate the risk of influenza for these patients. Physicians should consider alternative strategies such as vaccination of household contacts and prophylactic or early use of antiviral drugs to minimize the morbidity and mortality from influenza in this high-risk population.^33^

The primary studies included both the administration of one or two doses. From the pooled analysis the first vaccination induced a small response, and additional antibody was acquired after the second dose. Influenza vaccination of patients with hematological malignancies resulted in an adequate response, and the second vaccination induced additional antibody. It is therefore recommended to vaccinate this group twice.^25^

Two-dose vaccination was found to be superior to one dose, but it evoked only a limited serological response. Only half the patients achieved HI protective levels following two vaccinations, mainly those with low lymphocyte counts or allogeneic HSCT recipients having an unrelated donor. Therefore, post-exposure chemoprophylaxis with an adequate antiviral agent should also be given to providing maximal protection for HSCT recipients during influenza epidemics. To further decrease the risk of infection, HSCT patients’ household contacts and care providers should be immunized each season as well.^28^

As it regards the comparison between adjuvant and no adjuvant vaccine (H1N1) 2009 vaccine was safe and well tolerated and had a superior immunogenicity than that of the non-adjuvanted seasonal influenza vaccine. The use of adjuvanted vaccines may be the way to improve response to influenza vaccination in patients with hematological diseases.^24^

Rituximab is nowadays the most common anti-B cell biotherapy used in B-cell lymph proliferative disorders as a single agent or combined with chemotherapy; it is also used in many immunological diseases such as immune thrombocytopenia. Rituximab induces a deep depletion of normal B-cells leading to hypogammaglobulinemia. Many studies show that patients who receive or have recently received Rituximab have a very weak response, and often no response at all to influenza vaccine.^63^

Rituximab is a monoclonal antibody that specifically recognizes the CD20 antigen and induces phagocytosis of B cells. The CD20 antigen is expressed on malignant B cells and also on mature B cells. Therefore, administration of rituximab causes the destruction of malignant B cells as well as mature B cells, and persons under rituximab treatment show depletion of B cells. This type of B cell depletion can persist for long periods of time. It has been reported that even patients who had been in complete remission for long (≥6 mo) had low ability to induce antibodies against influenza vaccines. After receiving rituximab treatment, such patients do not attain the optimum antibody titer through influenza vaccination for a long time. The inhibitory effect of rituximab on antibody induction has been reported by many, but recently Ide Y et Al. demonstrated, through multivariate logistic regression analysis, that this effect is present also by eliminating the effect of the underlying disease (lymphoma),.^25^ None of the eleven patients receiving rituximab in addition to chemotherapy underwent seroconversion (p=0.05).^25^ It is important to be aware that such patients may fail to respond adequately to not only influenza vaccinations but also other common vaccines.^34^

Limitations of this study are related to some study does not mention adverse events, and the analysis did not consider pre-vaccination geometric mean titers. Repeated vaccinations could be useful.

Given acceptable immune response in patients with cancer and no reported serious adverse effect to the vaccine and taking into account the mortality and morbidity of influenza infection, clinicians can follow the AAP and ACIP recommendations for annual vaccination of children and immunosuppressed people.^64^,^65^

New vaccine border are the genetically engineered recombinant vaccines used in Safdar et al..^26^ Increasing concentrations of recombinant vaccine were associated with an increase in serum antibody responses against influenza A. Although the numbers were small, the highest given dose, 135 mg of each antigen, induced the highest frequency of responses for all 3 viruses. The results of this pilot study are consistent with our hypothesis that impaired antibody responses to influenza vaccine in patients with B-cell lymphoma may be improved by administering higher vaccine doses without significant reactogenicity.

### Pneumococcal Vaccine

There is few evidence from studies on the use of pneumococcal vaccination in adult and pediatric patients with hematological malignancies who underwent bone marrow transplantation or not. Most of the studies were old, nonrandomized, with a small number of patients, utilizing different immunization schedules and including in result also patient with non-neoplastic hematologic disorders. It is, therefore, difficult compare different patient groups and regimens. In this systematic review, we found trial and cohort studies include, in the same research, different type of vaccine and different type of patient.

In cohort studies, the authors found a worse response in hematologic patients than in healthy control or patients with other pathology. However, most agree in recommending the vaccine even after the bone marrow transplantation.

About the trials, mostly non-randomized, those on the effectiveness of 14-valent, dodeca-valent, 7 valent and 23 valent found in onco-hematologic subjects an impaired effectiveness than in healthy subject or people with other pathology. Except for the study of Patel in which patient received 2 doses of PCV7 followed by 1 dose of Pn-PS23 or 1 dose of Pn-PS23 followed by an additional dose of Pn-PS23. After administration of the booster dose of pneumococcal polysaccharide vaccine in previous conjugate recipients, very high concentrations against all PCV7 serotypes were achieved. In the study about 13 valent 53 CLL group and control group demonstrated a statistically significant increase in the IgG2 subclass levels after the vaccination. PCV13 vaccination in CLL patients is safe and induces an effective immune response in a considerable proportion of patients. To have an optimal vaccination response, the administration of PCV13 is recommended as soon as possible following CLL diagnosis.

A final thought is due to the role of healthcare workers in suggesting patients to get vaccinated and in performing these vaccinations on themselves to avoid a transmission to patients in their clinical practice. Sometimes, the health care workers have unclear attitude and behavior towards vaccinations, mainly due to low level of knowledge and exaggerated fears for side effects.^66^–^70^ This situation could be improved using both classical educational tools in the field of preventive medicine^71^–^72^ and new strategies that include new ways of communication on preventive strategies, as well as social marketing and social media.^73^–^75^

## Conclusions

Despite this low rate of response, flu, and pneumococcal vaccinations are likely still worthwhile for patients with hematological malignancies, as they are inexpensive interventions with few side effects. However, physicians and patients should be aware that vaccinations do not eliminate the risk of influenza and pneumococcal diseases for these patients. Physicians should consider alternative strategies such as vaccination of household contacts to minimize the morbidity and mortality from such diseases in this high-risk population. Prophylactic and early use of antiviral drugs could be considered as an additional preventive measure.^33^

Patients undergoing chemotherapy in particular rituximab, splenectomy, transplant recipient had lower and impaired response. No serious adverse events were reported for both vaccines.

## Figures and Tables

**Figure 2a:**
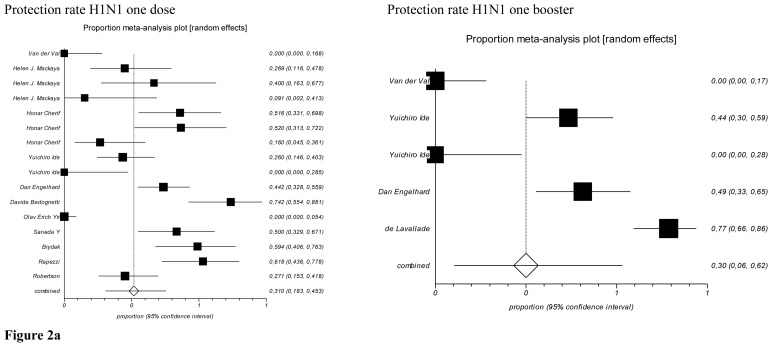
Forrest plots of Protection Rate stratify by a serotype of vaccine (Adults).

**Figure 2b:**
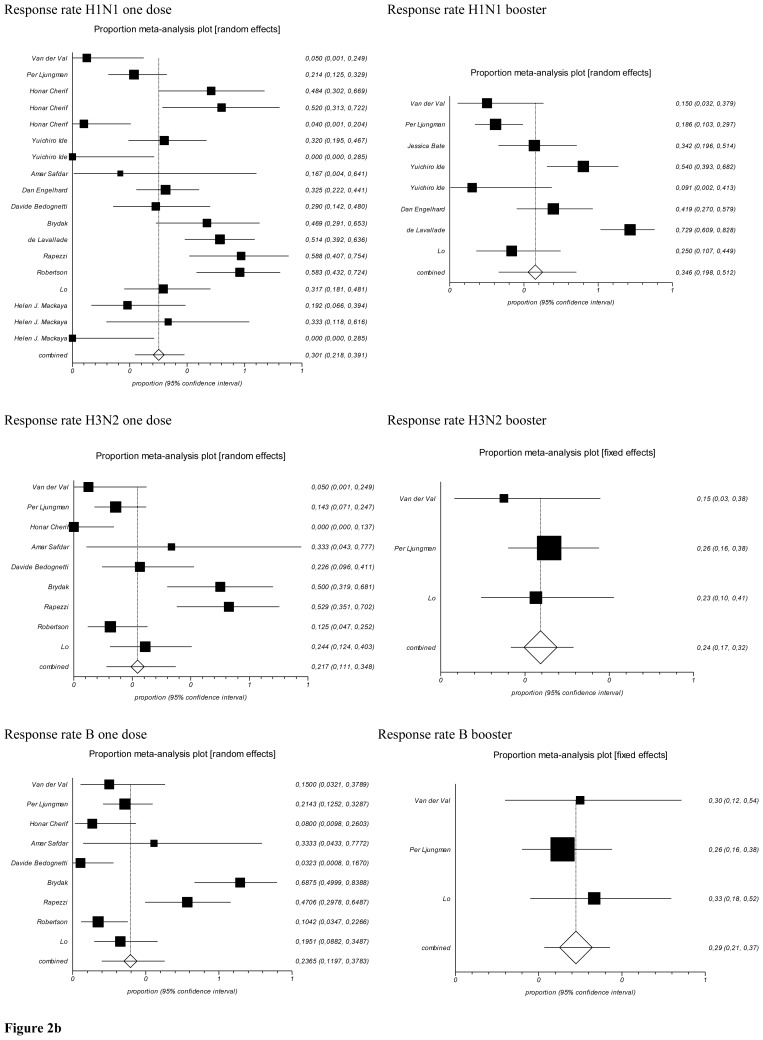
Forrest plots of Response Rate stratify by serotype of vaccine (Adults).

**Table 1:** characteristics of the studies included in the flu vaccination review.

First Author	Year of publication	Age (mean)	Diagnosis	Study design	Country	Vaccine	Quality score
van der Velden AMT	2001	71	B-CLL chronic lymphocytic leukaemia	cohort	The Netherlands	trivalent H1N1, H3N2, B 0.5 ml inactivated virus subunit vaccine	7
Ljungman P	2005	64	Monkmanhaematological malignancies	cohort	Stockholm, Sweden	trivalent inactivated influenza vaccine	5
Bate J	2010	6.3	tumor	cohort	England	split virion, AS03B-adjuvanted vaccine H1N1	7
Mackaya HJ	2010	56	solid and hematological malignancy	cohort	Toronto, ON, Canada	ASO 3-adjuvanated H1N1	7
Cherif H	2013	57	hematological diseases	cohort	Uppsala, Sweden	AS03-adjuvanted influenza A (H1N1) 2009 pandemic, vaccine H1N1 + trivalente	6
Ide Y	2014	59	hematological malignancy: Lymphoma, Acute Leukemia Myeloma, MDSa, Aplastic anemia	cohort	Osaka, Japan	a monovalent A(H1N1)pdm09 influenza vaccine	5
Safdar A	2006	55	Non-Hodgkin, B Cell Lymphoma	cohort	Houston, Texas	split-virus TIV or recombinant protein 15 mg, 45 mg, or 135 mg of each HA per 0.5-mL dose rHAO	6
Bedognetti D	2011	66	NHL patients in complete remission for ≥6 mo	cohort	Genoa, Italy	trivalent	8
Engelhard D	2011	50	55 allogeneic and 23 autologous hematopoietic stem cell transplantation (HSCT) recipients for hematological tumor	cohort	Beersheba, Israel	inactivated H1N1 v-like virus adjuvanted with AS03	6
Esposito S	2009	10	Onco-hematological disease, off therapy <6months, or 6–24 months	cohort	Milan, Italy	trivalent	8
Yri OE	2016	63	lymphoma patients	cohort	Stavanger, Norway	H1N1	7
Porter CC	2004	7,7	Acute Lymphoblastic Leukemia	cohort	Nashville, Tennessee	inactivated trivalent influenza vaccine	7
Shahgholi E	2010	11	ALL on maintenance therapy	cohort	Tehran, Iran	trivalent inactivated influenza vaccine	7
Sanada Y	2016	61	haematological cancer	cohort	Japan	trivalent vaccine H1N1 H3N2 B	6
Brydak LB	2006	>20	haematological cancer	cohort	Poland	trivalent vaccine H1N1 H3N2 B	6
de Lavallade H	2011	adults	haematological cancer	cohort	England	inactivated split-virion 3.75 μg of hemagglutinin and AS03 adjuvant (Pandemrix GSK) H1N1 H3N2 hemagglutinin and AS03 adjuvant (Pandemrix GSK, UK)	7
Rapezzi D	2002	adults	haematological cancer	cohort	Italy	Inflexal V Vaccine H1N1 H3N2 B	6
Robertson JD	2000	adults	haematological cancer	cohort	England	Trivalent Fluvirin (Evans Medical)	7
Lo W	1993	adults	haematological cancer	cohort	USA	trivalent influenza split-virus vaccine	5
Monkman K	2011	66.5	hematological malignancies patients	CT	Italy	ArepanrixTM split influenza virus, A/California/7/2009 (H1N1)v-like strain, and ASO3, an oil-in-water adjuvant	1
Dignani MC	2015	51	patients (47) had onco-hematological cancers and (18) had solid tumors.	CT	Argentina	seasonal trivalent influenza vaccine	0
Dotan A	2014	0–18	haematological cancer	cross-sectional	Israel	Pandemrix—influenza vaccine (H1N1) (split virion, inactivated, adjuvanted) vaccine (H1N1) (split virion, inactivated, adjuvanted)	5

**Table 2:** Description of adverse events (AEs) for flu vaccination, reported in the studies selected.

First author	Year of publication	Local reaction	Systemic reaction	adverse events (AEs)
Mackaya HJ	2010	23/41	1 (mild fatigue)/41	0
Monkman K	2011			
Cherif H	2013	Nr	Nr	0/25
Ide Y	2014	Nr	Nr	0/50
Safdar A	2006	6/6	0/6	0/6
Esposito S (off therapy <6 months)	2009	4/67	25/67	0/67
Esposito S (off therapy 6–24 months)	2009	4/65	26/65	0/65
Porter CC	2004	Nr	Nr	0/60
Shahgholi E	2010	0/35	0/35	0/35

**Table 3:** Characteristics of cohort studies of pnemococcal vaccine included in the review.

First Author	Year	Country	Age	Diagnosis	Vaccine	Timing	Doses	Quality score
Braconier JH	1984	Denmark	spletectomized: mean age 52,6 - non splenectomized: mena age 42.5	Patients with malignant hematologic diseases (spletectomized and non-splectomized)	14-valent	Not specified	Not specified	4/6
Cherif H	2006	Sweden	median age at inclusion was 52 years (range 18–82 years)	Splenectomized individuals with hematological disorders	Pneumovax 23	Antibody levels measured 1 year after vaccine. who has antibody level <0.7 were revaccinated After 5 years, all individuals were revaccinated	Not specified	6/6
Pao M	2008	Usa	23 median age. Children 9 years median) and adult median age : 41	Acute Leukemia, chronic Leukemia, aplastic Anemia, NHL	Prevenar 7 most of patient pneumovax to patient as first vaccine	median time 1.1 years after HCT.	No serious adverse effect	4/6
Meerveld-Eggink A	2009	Netherlands	42–67	Patient with various hematologic malignant disease	Prevenar, pneumovax	1 year + 1 week after transplantation	Not specified	4/6
Cheng FWT	2012	China	between 1 year and 18 years	Cancer patients who had received intensive chemotherapy for haematological malignancies or solid tumours	PCV-7	Two doses of PCV-7 were given 4 weeks apart.	0.5 ml	5/6
Hinge M	2012	Denmark	Age [median (range) years]: 57.8 non-responders; 56.2 responder	Patients with multiple myeloma	23-valent	The patients were vaccinated against S. pneumoniae at the time of peripheral stem cell harvest	Not specified	6/6
Llupià A	2012	Spain	patients aged >16 years	Patient included in the protocol for splenectomised patients for ematologic malignancies)	Pneumo 23	from 15 days before to 15 days after surgery	Not specified	4/6
Shah GL	2015	Usa	34 median age	CBT (cord blood transplant recipients )treated for hematological malignancies (predominantly had acute leukemia	Prevenar 7 Prevenar 13	Patient were vaccinated at a median of 17 months post-CBT	Not specified	3/6

**Table 4:** Characteristics of the trial included in the review on pneumococcal vaccine.

First Author	Year	Country	Age	Study population	Vaccine	Timing	Doses	Quality score
Siber GR	1978	USA	Range 7–57 years	Cases: all patients had completed therapy for Hodgkin’s disease clinical remission. Controls: selected from the families of the patients or the hospital staff	12 valent	Not specified	0,5 ml	**0/3**
Feldman S	1985	USA	from 3 to 11 year	Pt with acute lymphocytic leukemia in continuous complete remission	14 valent	Children were vaccinated at 1, 3 or 6 months following induction of initial remission or 4–6 weeks following cessation of 30 months of antileukemia therapy	0.5 ml	**0/3**
Frederiksen B	1989	Denmark	37,4 mean age	Non-splenectomized volunteers, patients with HD	14 valent pneumoc vaccine	Group 1 and 2: vaccinated 1 wk, 2 months before splenectomy and therapy. Group 3: vaccinated during or after having received treatment Group 4 vaccinated after splenectomy and radiotherapy Group 5: vaccinated after splenectomy and radio-plus chemotherapy with a mean interval of 3 1/12 yr	50 μg	0/3
Chan CY	1996	USA	Mean age: primed 39 unprimed 38	Patients with HD 1969 – 1991 who had not relapsed or developed second tumors	7-valent 23-valent	HD patients who receive 7-valent coniugate vaccine were offered 23-PS vaccine 1 year later, 39 patients agreed. Other HD patients receive 23-PS vaccine only in the initial study.	Not specified	**0/4**
Parkkali T	1996	Finland	>16 years	Hematologic malignancies	23 valent	Late group ( 18 month) and early group (6 months after BMT.	Not specified	0/3
Petrasch S	1997	Germany	Median age: 52 lymphoma patients and 45 patients with nonneoplastic diseases	Splenectomized, unvaccinated patients with B-cell non Hodgkin disease and patients who had undergone splenectomy for other reasons	23-valent pneumococcal capsular polysaccharide vaccine.	Immunization immediately prior or subsequent to splenectomy. Patients who did not respond to the primary vaccination and patients who were in remission received a 0.5-ml booster dose of the vaccine. All patients receiving a second immunization	0.5 ml	**0/3**
Robertson JD	2000	UK	55.4 years	Multiple myeloma patient	Pneumovax II 23-valent	Three vaccination at non specified interval	Not serious adverse reaction	**0/3**
Gandhi MK	2001	Uk	mean ages 47.4 (autoPBSCT), 34.9 (autoBMT), and 40.7 (alloBMT).	AutoPBSCT, autoBMT and alloBMT patient	non-conjugated polysaccharide 23-valent	Mean time of vaccination following SCT was: 11 months for autoPBSCT 12 months for autoBMT 16 months for alloBMT	Not serious adverse reaction	**0/3**
Hartkamp A	2001	Netherland	70.4	B cell chronic lymphocytic leukaemia patient	Pneumovax-23	The patients received simultaneously injection of a 23-valent pneumococcal polysaccharide vaccine	0,5 ml	**1/3**
Nordøy T	2001	Norway	median age : 39.5 years and 39 Healthy blood donors	Patients with malignant lymphoma years after ABMT	Pneumovax	All patients and controls received one vaccination against pneumococcal disease.	Not specified	**0/3**
Sinislao M	2001	Finland	mean age 66	31 patients CLL and 25 controls	Pnu-Immune 23	Not specified	0,5 mL	**0/3**
Nordøy T	2002	Norway	20–75 year	Patient with Solid tumors and malignant lymphoma patient undergoing chemotherapy	23 valent and influenza	All patients were vaccinated between two courses of chemotherapy	Not specified	**0/3**
Landgren O	2004	Sweden	28 years	Patients with : HL (Hodking linfoma), autoimmune haemolytic anaemia (AIHA), thrombocytopenic purpura (ITP), splenectomy due to splenic rupture caused by trauma	Pneumovax 23 valent	All patients were immunized. They were revaccinated depending on their individual PS antibody levels. After 5 years, all individuals were revaccinated	25 μg of capsular PS from each of 23 pneumococcal serotypes	**0/3**
Antin JH	2005	USA	>2 years	Patients older than 2 years of age with an diagnosis of a hematologic malignancy and who were scheduled to receive an autologous stem cell transplant.	PCV7 conjugate vaccine	Patients receive a dose PCV7 7 to 10 days before stem cell collection or no vaccine before stem cell collection. After reinfusion of stem cells all study patients were immunized with PCV7 at 3, 6, and 12 months.	Not Specified	**0/4**
Eigenberger K	2007	Austria	adult	Splenectomized adult patients suffered from hematological malignancies and patients were splenectomized following trauma	Pneumo 23	HM group, 9 patients received chemotherapy within 3 months prior to vaccination and 2 patients were vaccinated before splenectomy.	0.5 ml	**0/3**
Patel SR	2007	UK	aged 1–18 years.	All patients had undergone HSCT for underlying malignancies	Prevenar, Pneumovax	Revaccination 12 months after autologous and HLA-identical sibling HSCT and >18 months after any other allogeneic HSCT. 2 schedules: (1) administration of PCV7 at 15 and 16 months after transplantation and administration of PnPS-23 at 24 months after transplantation, and (2) administration of PnPS-23 at 15 and 24 months after transplantation. The scheduled administrations were started 6 months later for “other” allogeneic HSCT.	Not specified	**0/4**
Sinislao M	2007	Finland	65 years median	Chronic lymphocytic leukemia (CLL), control	7-valent pneumococcal conjugate vaccine	Not specified	0.5 ml	**0/3**
Pasiarski M	2014	Poland	Mean age 66 CLL and 68.6 control	Untreated patients with CLL control group 15 healthy, age- and sex-matched individuals	Prevenar 13	The mean follow-up period from the time of vaccination was median: 20.75 months	No adverse reaction	**0/3**
